# Immunohistochemical validation of COL3A1, GPR158 and PITHD1 as prognostic biomarkers in early-stage ovarian carcinomas

**DOI:** 10.1186/s12885-019-6084-4

**Published:** 2019-09-18

**Authors:** Hanna Engqvist, Toshima Z. Parris, Anikó Kovács, Szilárd Nemes, Elisabeth Werner Rönnerman, Shahin De Lara, Jana Biermann, Karin Sundfeldt, Per Karlsson, Khalil Helou

**Affiliations:** 1 Department of Oncology, Institute of Clinical Sciences, Sahlgrenska Cancer Center, Sahlgrenska Academy at University of Gothenburg, Gothenburg, Sweden; 2 Department of Clinical Pathology, Sahlgrenska University Hospital, Gothenburg, Sweden; 3 Department of Orthopaedics, Institute of Clinical Sciences, Sahlgrenska Academy at University of Gothenburg, Gothenburg, Sweden; 4 Department of Obstetrics and Gynecology, Institute of Clinical Sciences, Sahlgrenska Academy at University of Gothenburg, Gothenburg, Sweden

**Keywords:** Ovarian cancer, Prognostic biomarker, Immunohistochemistry, Mucinous ovarian cancer, Clear-cell ovarian cancer

## Abstract

**Background:**

Ovarian cancer is the main cause of gynecological cancer-associated death. However, 5-year survival rates differ dramatically between the five main ovarian carcinoma histotypes. Therefore, we need to have a better understanding of the mechanisms that promote histotype-specific ovarian carcinogenesis and identify novel prognostic biomarkers.

**Methods:**

Here, we evaluated the prognostic role of 29 genes for early-stage (I and II) ovarian carcinomas (*n* = 206) using immunohistochemistry (IHC).

**Results:**

We provide evidence of aberrant protein expression patterns for Collagen type III alpha 1 chain (COL3A1), G protein-coupled receptor 158 (GPR158) and PITH domain containing 1 (PITHD1). Kaplan-Meier survival analysis revealed that COL3A1 expression was associated with shorter overall survival in the four major histotypes of epithelial ovarian carcinoma patients (*P* value = 0.026, HR = 2.99 (95% CI 1.089–8.19)). Furthermore, GPR158 and PITHD1 were shown to be histotype-specific prognostic biomarkers, with elevated GPR158 expression patterns in mucinous ovarian carcinoma patients with unfavorable overall survival (*P* value = 0.00043, HR = 6.13 (95% CI 1.98–18.98)), and an association with lower PITHD1 protein expression and unfavorable overall and disease-specific survival in clear-cell ovarian carcinoma patients (*P* value = 0.012, HR = 0.22 (95% CI 0.058–0.80); *P* value = 0.003, HR = 0.17 (95% CI 0.043–0.64)).

**Conclusions:**

The novel biomarkers identified here may improve prognostication at the time of diagnosis and may assist in the development of future individualized therapeutic strategies for ovarian carcinoma patients.

**Electronic supplementary material:**

The online version of this article (10.1186/s12885-019-6084-4) contains supplementary material, which is available to authorized users.

## Background

Ovarian cancer is the most lethal gynecological cancer with a five-year survival rate of about 55% in Sweden and 47% in the US [1, 2]. Epithelial cancers account for about 90% of all ovarian cancers and are distributed over the most common histotypes: high-grade serous (HGSC, 70%), low-grade serous (LGSC, < 5%), endometrioid (EC, 10%), mucinous (MC, 3–4%) and clear-cell ovarian carcinomas (CCC, 10%) [3]. Five-year survival rates differ significantly across the histotypes, with drastically lower survival rates for serous carcinoma (SC (HGSC and LGSC), 43%) compared to EC (82%), MC (71%) and CCC (66%) in the US. This is in line with the high number of patients (80%) with SC that are diagnosed at advanced stages (stages III and IV) as well as EC, MC and CCC that are predominantly diagnosed at stage I (58–64%) [4]. Hence, in view of the diverse survival rates for the different histotypes, it is crucial to identify novel prognostic biomarkers for each histotype.

Cancer antigen 125 (CA 125) is routinely used in the clinic for, e.g. preoperative diagnosis, to monitor response to chemotherapy, disease progression and relapse of epithelial ovarian cancer [5]. However, it may not be suitable for the detection of early-stage ovarian cancer nor the MC histotype [5–7]. Additional serum biomarkers, such as CA 19–9, CA 15–3, CA 72–4 and CEA are also routinely used in clinical practice [8]. Biomarker panels, such as CA 125 in combination with human epididymis protein 4 (HE4) (Risk of Ovarian Malignancy Algorithm, ROMA) and the multivariate index assay (MIA2G) comprising CA 125, transferrin, apolipoprotein A-1, follicle-stimulating hormone and HE4 (Overa), have been approved by the US Food and Drug Administration (FDA) for preoperative testing to determine the likelihood of malignancy [9, 10]. Apart from the use of PARP inhibitors, no prognostic biomarkers for the management of ovarian cancer are currently used in the clinic. Some recent reports have evaluated prognosis in relation to histotype, e.g. the expression of cancer antigen 45 (CT 45) was shown to be a prognostic factor for positive response to platinum-based chemotherapy in HGSC [11]. Furthermore, high expression of aquaporin-1 (AQP1) in MC and EC, and low expression of AQP1 in CCC were associated with unfavorable prognosis [12].

Early-stage epithelial ovarian cancers generally have a more favorable prognosis with an overall five-year survival rate of 89% for stage I and 71% for stage II [4]. Yet around 16% of stage I and II ovarian cancer patients have worse prognoses [4]. Hence, it is important to identify novel biomarkers for use in ovarian cancer prognostication to aid in the management of ovarian cancer in order to identify patients with aggressive disease. In view of differences between the histotypes, histotype-based gene panels for prognosis are also needed to guide therapeutic decisions. In the current study, immunohistochemistry (IHC) was used to evaluate the clinical relevance of 29 promising prognostic biomarkers (ARHGAP21, ARMC3, C7, CDH18, CES3, COL11A1, COL3A1, EHD3, FRMPD2, GABRP, GID4, GPR158, GRM5, IGHG1, JCHAIN, KIF26B, MAP7D2, MTRNR2L1, MTUS1, MUC15, PITHD1, PTEN, RTKN2, SLC9A4, SMYD2, TRIM71, TRIO, TTK, and VNN1) for early-stage ovarian carcinoma identified using RNA sequencing (RNA-seq) data. In our previous work, *MTUS1* frameshift insertion was associated with differences in gene expression and overall survival, and *COL3A1* gene expression were shown to correlate with tumor aggressiveness [13]. The remaining 27 biomarkers were identified using Cox regression models to correlate gene expression data (RNA-seq) with survival status.

## Methods

### Patients and tumor samples

Full-face formalin-fixed paraffin-embedded (FFPE) specimens were obtained from the Departments of Clinical Pathology at hospitals in Western Sweden for 206 early-stage (stage I and II) primary invasive ovarian carcinoma patients diagnosed between 1994 and 2006, of which 95 samples corresponded to fresh-frozen tumor samples previously analyzed by RNA-seq [13]. All tumor specimens were reclassified according to current WHO criteria [14–17] by pathologists at Sahlgrenska University Hospital. Further clinicopathological characteristics data were obtained from the National Quality Registry at the Regional Cancer Center West (Gothenburg, Sweden) and the Cancer Registry at the National Board of Health and Welfare (Table 1). All procedures were performed in accordance with the Declaration of Helsinki and approved by the Regional Ethical Review Board (Gothenburg, Sweden; case number 767–14). The Regional Ethical Review Board approved a waiver of written consent to use the tumor specimens.

**Table 1:** Clinicopathological characteristics for the 206 ovarian carcinoma patients

	Number of patients (%)				
	HGSC	EC	MC	CCC	
	(n = 94)	(n = 46)	(n = 29)	(n = 37)	*P* value
Patient age					0.457
Mean	64	62	60	65	
Range	22–88	25–83	30–82	42–84	
Overall Survival					0.082
0-2y	7 (7)	3 (7)	6 (21)	5 (14)	
2-5y	26 (28)	9 (20)	3 (10)	10 (27)	
5-10y	28 (30)	7 (15)	7 (24)	8 (22)	
>10y	33 (35)	27 (59)	13 (45)	14 (38)	
Cause of death					0.001
Ovarian carcinoma	53 (56)	7 (15)	5 (17)	19 (51)	
Other cancer	8 (9)	6 (13)	4 (14)	2 (5)	
Other	10 (11)	10 (22)	8 (28)	6 (16)	
Alive	15 (16)	17 (37)	7 (24)	8 (22)	
Not available	8 (9)	6 (13)	5 (17)	2 (5)	
Stage					0.005
I	51 (54)	32 (70)	22 (76)	31 (84)	
II	43 (46)	13 (28)	7 (24)	6 (16)	
Tumor grade EC					NA
FIGO grade I	NA	11 (24)	NA	NA	
FIGO grade II	NA	27 (59)	NA	NA	
FIGO grade III	NA	8 (17)	NA	NA	
Dualistic model					0.643
Type I	0 (0)	46 (100)	29 (100)	37 (100)	
Type II	94(100)	0 (0)	0 (0)	0 (0)	
CA125					0.106
<35	17 (18)	13 (28)	10 (35)	14 (38)	
35–65	17 (18)	7 (15)	8 (28)	8 (22)	
>65	60 (64)	25 (54)	11 (38)	15 (41)	
Not available	0 (0)	1 (2)	0 (0)	0 (0)	
Ploidy					0.168
Near diploid	22 (23)	17 (37)	7 (24)	5 (14)	
Aneuploid	69 (73)	26 (57)	19 (66)	30 (81)	
Not available	3 (3)	3 (7)	3 (10)	2 (5)	
Chemotherapy					0.212
Yes	91 (97)	42 (91)	27 (93)	37 (100)	
No	0 (0)	0 (0)	0 (0)	0 (0)	
Not available	3 (3)	4(9)	2 (7)	0 (0)	

### Selection of study genes

Raw RNA-seq read counts (log2-values) for 95 of the 206 ovarian tumors were used to correlate gene expression data with survival status (overall survival (OS) and disease-specific survival (DSS)) for each histotype (HGSC, EC, MC, CCC) using univariable Cox proportional hazards models and the Benjamini–Hochberg procedure to control the false discovery rate (FDR) with FDR-corrected *P* values < 0.05 [13]. The Cox regression analysis assumes the following relationship between the baseline and observed hazard *h*_1_(*t*) = *h*_*o*_ (*t*)*e*^∑*βx*^. Here, *e*^*βx*^ quantifies the effect of individual probes on the baseline hazard. Using the output from the Cox regression analysis, an index was defined as *E*{*x*| *βx* > 0} − *E*{*x*| *βx* < 0}, wherein *E* is the expected value. The index assessed the absolute difference in mean log2 ratio of different probes among patients with favorable *βx* < 0 and unfavorable *βx* > 0 prognoses. The predictive power of the regression models was assessed with time-dependent Area under the receiver operating characteristics curve [(AUC(t)] values and summarized as the concordance index (C-index) for survival data [18]. The C-index varies between 0.5 (random ordering of the survival time with no predictive power) and 1 (perfect ordering of the survival times, i.e. perfect prediction of survival times). Twenty-seven genes, henceforth termed study genes, were selected among genes with *P* values below 0.05 and the highest C-index and/or the highest absolute log2 ratio value.

### Immunohistochemical analysis

Four micrometer FFPE sections were prepared on Dako FLEX IHC microscope slides and dried in an oven for 1 hour at 60 °C. Antibodies for the study proteins were primarily chosen from the Human Protein Atlas (HPA) [19, 20]. Optimal antibody dilutions were achieved using an optimization panel consisting of 15 full-face FFPE ovarian carcinoma sections representing varying histotypes (HGSC, EC, MC, CCC) and International Federation of Gynecology and Obstetrics (FIGO) stages. If an antibody dilution of 1:25 resulted in weak or no staining, no further dilutions were tested. One sample in the optimization panel was chosen as positive control for each immunohistochemical experiment. Immunostaining was performed for each protein using the optimized antibody dilutions (Table 2). The sections were immunostained on a Dako Autostainer Plus (Agilent Technologies) using Dako EnVision FLEX visualization systems. More specifically, deparaffinization and antigen retrieval were performed using EnVision FLEX high pH target retrieval solution (pH 9), the sections were stained using liquid DAB (3,3′-diaminobenzidine) 2-component system, and subsequently counterstained with EnVision FLEX hematoxylin (link). After immunostaining, the sections were rinsed with deionized water, dehydrated in an ethanol series comprised of 70, 95 and 100% ethanol, cleared in xylene and mounted.

**Table 2:** Statistical characteristics for the study genes and selected antibodies and corresponding optimized antibody dilution factors for IHC experiment. Twenty-seven genes are listed in view of their respective HR, 95% CI, *P* value, C-index and log2 ratio. *MTUS1* and *COL3A1* were selected in view of the findings in our previous work. An optimized dilution factor for IHC analysis could be determined for 12 of the 29 proteins. All antibodies with determined optimized dilution were polyclonal antibodies except the antibody for PTEN which was monoclonal

Gene symbol	Histotype	Survival	HR	95% CI	*P* Value	C-index	Log2 ratio	Antibody	Company	Optimized dilution
*ARHGAP21*	EC	OS	1.00	1.00–1.003	0.0011	0.80	1.53	22,183–1-AP	Nordic Biosite	1:50
*ARMC3*	HGSC	OS	0.80	0.70–0.92	0.0013	0.64	−3.83	HPA037823	Sigma-Aldrich	-
*C7*	EC	OS	1.32	1.03–1.69	0.0310	0.72	5.55	HPA001465	Sigma-Aldrich	-
*CDH18*	HGSC	OS	1.18	1.06–1.32	0.0020	0.64	5.67	HPA014416	Sigma-Aldrich	-
*CES3*	EC	OS	0.66	0.49–0.90	0.0088	0.74	−4.17	HPA041008	Sigma-Aldrich	1:500
*COL11A1*	CCC	OS	1.49	1.13–1.96	0.0042	0.77	4.32	ab64883	Abcam	-
*EHD3*	CCC	DSS	11.67	2.23–59.35	0.0031	0.95	2.50	HPA049890	Sigma-Aldrich	-
*FRMPD2*	EC	OS	0.76	0.60–0.96	0.0228	0.71	−5.34	HPA045059	Sigma-Aldrich	-
*GABRP*	CCC	OS	1.34	1.03–1.73	0.0275	0.71	5.05	PA5–46830	Thermo Fisher	-
*GID4*	EC	OS	1.04	1.02–1.07	0.0013	0.79	0.75	HPA044348	Sigma-Aldrich	1:150
*GPR158*	MC	OS	1.44	1.02–2.04	0.0380	0.77	4.93	HPA013185	Sigma-Aldrich	1:25
*GRM5*	EC	OS	0.56	0.35–0.91	0.0179	0.73	−4.17	ab76316	Abcam	-
*IGHG1*	CCC	DSS	1.20	1.04–1.39	0.0128	0.72	7.40	SAB1401207	Sigma-Aldrich	1:25
*JCHAIN*	CCC	OS	1.23	1.04–1.46	0.0133	0.73	6.34	HPA044132	Sigma-Aldrich	-
*KIF26B*	EC	DSS	0.46	0.23–0.91	0.0248	0.91	−3.68	HPA027709	Sigma-Aldrich	-
*MAP7D2*	MC	OS	1.86	1.09–3.16	0.0218	0.83	4.06	HPA051508	Sigma-Aldrich	-
*MTRNR2L1*	HGSC	OS	1.23	1.08–1.40	0.0017	0.64	5.68	HPA059729	Sigma-Aldrich	-
*MUC15*	EC	OS	0.66	0.50–0.87	0.0032	0.79	−5.33	HPA026110	Sigma-Aldrich	-
*PITHD1*	CCC	DSS	146.53	6.19–3470	0.0020	0.89	0.57	PAB20914	Abnova	1:50
*PTEN*	EC	DSS	404.90	1.23–133,809	0.0425	0.96	0.99	ab109454	Abcam	1:100
*RTKN2*	MC	OS	7.87	1.48–41.96	0.0157	0.89	1.66	17,458–1-AP	Nordic Biosite	1:25
*SLC9A4*	MC	OS	0.65	0.44–0.96	0.0292	0.76	−4.65	HPA036096	Sigma-Aldrich	-
*SMYD2*	CCC	DSS	56.79	3.92–822.37	0.0031	0.89	0.91	PA5–51339	Thermo Fisher	-
*TRIM71*	HGSC	DSS	0.78	0.66–0.92	0.0036	0.67	−4.30	HPA038141	Sigma-Aldrich	-
*TRIO*	CCC	DSS	19.72	2.47–157.47	0.0049	0.93	1.27	HPA008157	Sigma-Aldrich	1:25
*TTK*	MC	OS	5.76	1.52–21.83	0.0100	0.88	1.98	PAB3320	Abnova	1:25
*VNN1*	CCC	OS	1.36	1.05–1.75	0.0184	0.71	4.48	HPA064145	Sigma-Aldrich	-
*COL3A1*	95 RNA-seq samples	OS	-	-	-	-	-	HPA007583	Sigma-Aldrich	1:50
*MTUS1*	95 RNA-seq samples	OS	-	-	-	-	-	ab198176	Abcam	1:40

### Determination of immunoreactive score

Microscopic evaluation of immunostained tissue sections was performed by two pathologists (AK and EWR; blinded to the survival data) based on the percentages and staining intensity (weak, moderate, strong) in tumor cells, as well as the staining intensity in peritumoral stromal and normal cells, to distinguish protein staining intensities in different cell types within the tumor. An immunoreactive score (H-score) was calculated for each tumor specimen based on the percentage and intensity of positively stained tumor cells, where 0 = negative, 1 = weak positive, 2 = moderate positive and 3 = strong positive staining. The H-score values ranged between 0 and 300, where H-score equaled (1 x %1) + (2 x %2) + (3 x %3) [21]. The H-score was used to correlate the protein expression levels to OS and DSS. An H-score cutoff stratifying the tumor specimens in positive and negative protein expression was determined for each protein using Kaplan-Meier plots in X-tile Software (v. 3.6.1) [22].

### Statistical analysis

Statistical analyses were performed using *P* values < 0.05 (two-sided) in R/Bioconductor v. 3.5.1. Univariable and multivariable Cox proportional hazard models were calculated for COL3A1, GPR158 and PITHD1 expression in relation to OS (defined as the time from initial diagnosis to death from any cause) and DSS (defined as the time from initial diagnosis to ovarian cancer-related death) using the 95 RNA sequenced samples for COL3A1, MC samples for GPR158, and CCC samples for PITHD1. Kaplan-Meier curves were generated and tested with log rank tests using survival time and dichotomized H-score for positive immunostaining (survival v. 2.40–1, survminer v. 0.4.3) [23, 24]. The relationship between clinicopathological parameters and positive/negative protein expression were calculated using two-tailed Fisher’s exact test (tableone v. 0.9.3) [25]. Multivariable Cox proportional hazard models were used to assess the predictive strength (C-index) of COL3A1, GPR158 and PITHD1 when adjusted by established clinical parameters (age, stage, CA125, ploidy and/or histotype). Box plots were generated to compare RNA-protein expression and differences in H-score between the histotype and survival groups using ggplot2 (v. 3.1.0) and Kruskal-Wallis test [26]. For external validation, the Kaplan-Meier (KM) plotter online tool (http://kmplot.com/analysis/) for ovarian cancer (*n* = 1657) was used to determine the clinical relevance of gene expression for the study genes in relation to OS [27]. The REMARK reporting recommendations for prognostic biomarkers were applied to this study (Additional file 5: Table S1) [28].

## Results

### Selection of study genes with prognostic value

To identify genes with prognostic value for each histotype, Cox regression models were used to generate genetic signatures with raw RNA-seq read counts in relation to survival data (OS, DSS). In total, 2223 and 2261 genes with *P* values < 0.05 were identified in HGSC for OS and DSS. For EC, 1440 and 522 genes, and 3557 and 1827 genes for CCC were associated with OS and DSS. For MC, 970 genes were significantly associated with OS. Nine promising prognostic genes were selected among the top 10 genes with the highest C-index (Table 2). A further selection of genes was additionally based on log2 ratio. In HGSC, 670 and 1278 genes with *P* values < 0.05, C-index > 0.6 and were identified for OS and DSS. With regard to EC, 512 (OS) and 521 genes (DSS), 1080 (OS) and 1501 genes (DSS) for CCC, and 885 genes (OS) for MC were associated with prognosis (*P* values < 0.05, C-indices > 0.7). Eighteen promising prognostic genes were selected among the top 20 genes with the highest log2 ratio (log2 ratio > |3.8| for HGSC, log2 ratio > |3.5| for EC, MC, CCC) (Table 2). The selection of biomarkers to be tested with IHC was also based on high gene expression levels and a variation in gene expression depending on histotype-specific survival rates. The previously identified genes, *MTUS1* and *COL3A1*, wherein carriers of MTUS1 frameshift insertion associated with a significant difference in gene expression and OS, and COL3A1 gene expression correlated with tumor aggressiveness, were also included in the selection [13].

### IHC analysis identified histotype-specific aberrant protein expression patterns

Optimized antibody dilutions could be determined for 12 (ARHGAP21, CES3, GID4, GPR158, IGHG1, PITHD1, PTEN, RTKN2, TRIO, TTK, COL3A1, and MTUS1) of the 29 proteins using the optimization panel (Table 2). Seventeen antibodies had either no or too weak staining at 1:25 and were therefore not studied further. IHC was used to evaluate COL3A1 and MTUS1 protein expression levels in the 95 RNA sequenced samples, whereas the remaining 10 proteins were examined in respective histotypes (Table 2). ARHGAP21 and IGHG1 were strongly expressed in both stromal and tumor cells, and therefore excluded from further study. Positive staining was interpreted as H-score > 20 for COL3A1, > 0 for GPR158, and > 60 for PITHD1, while no significant H-score cutoff could be determined for CES3, GID4, MTUS1, PTEN, TRIO and TTK. However borderline significance was found for RTKN2 protein expression in MC samples in relation to OS (*P* value = 0.054, HR = 3.94 (0.89–17.50), H-score cutoff > 90).

**Fig. 1:**
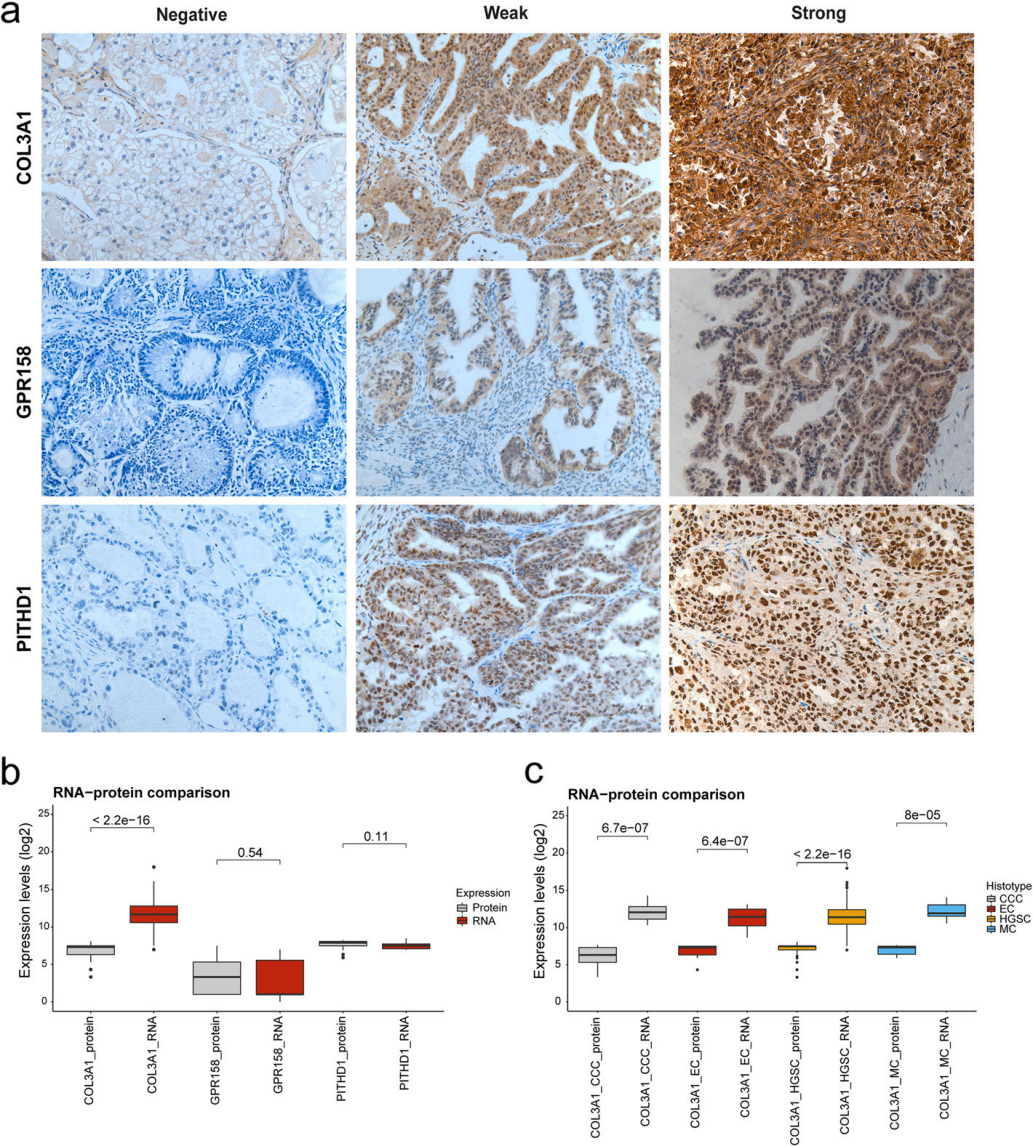
COL3A1, GPR158, PITHD1 protein expression in ovarian tumor cells and comparison between RNA and protein expression**.** a) shows representative IHC staining intensities (negative-strong) in hot spots for COL3A1, GPR158 and PITHD1 (200 x magnification). COL3A1 was shown to be negative, weak and strong in the CCC, EC and HGSC histotypes. Box plots illustrating the comparison between the distributions of log2 values of raw RNA-seq read counts and H-score for COL3A1, GPR158 and PITHD1, with b) all H-score values are taken into account. Similar RNA and protein expression is shown for GPR158 and PITHD1 (GPR158 *P* value = 0.54, PITHD1 *P* value = 0.11). For COL3A1, higher RNA expression in comparison with protein expression was revealed. This was also seen in c) when stratified by histotype

Generally, the immunostaining patterns for COL3A1, GPR158 and PITHD1 were homogeneous with the exception of two samples (the staining intensity for one COL3A1 sample and one GPR158 sample were evaluated as weak-moderate staining). COL3A1 protein expression varied in the 95 RNA sequenced ovarian carcinoma samples. The IHC results showed that the COL3A1 protein was mainly localized to the cytoplasm of tumor cells (Fig. 1a) and the vast majority of samples were COL3A1-positive (*n* = 87, 92%). Positive COL3A1 staining was detected in all histotypes, whereas negative staining was not detected in MC. GPR158 immunostaining was evaluated in the MC histotype and was found to show GPR158-positivity in 17/29 patients (59%). The GPR158 protein was mainly localized to the cytoplasm of tumor cells, but occasional staining was also found in tumor cell nuclei. Finally, PITHD1-positivity was found in the vast majority of the CCC samples (*n* = 34, 92%), with positive immunostaining primarily observed in tumor cell nuclei (Fig. 1a). In addition, slight to moderate expression of PITHD1 were also seen in stromal cells. No association was found between COL3A1, GPR158 or PITHD1 protein expression and clinicopathological characteristics (Additional file 6: Table S2). COL3A1 expression was predominantly observed as intermediate staining, GPR158 as weak staining and PITHD1 as strong staining (Additional file 1: Figure S1). The H-score for COL3A1 expression varied depending on histotype, with low COL3A1 expression levels in CCC samples (*P* value = 0.0015, Additional file 2: Figure S2a), particularly samples in the 5–10 year survival group (*P* value = 0.001, Additional file 2: Figure S2b).

The H-score data was further compared with the raw RNA-seq read counts, by converting both datasets to log2 values. For GPR158 and PITHD1, two analyses were performed with 1) comparing RNA-seq read counts for the 95 RNA sequenced samples with H-score values for the patient cohort, and 2) comparing RNA-seq read counts with corresponding H-score values within the 95 RNA sequenced samples. For both analyses, no difference was found between the RNA and protein expression patterns for GPR158 and PITHD1 (first analysis: GPR158 *P* value = 0.54, PITHD1 *P* value = 0.11, second analysis: GPR158 *P* value = 0.66, PITHD1 *P* value = 0.68). COL3A1 protein expression was lower than *COL3A1* RNA expression, which was also seen when stratified by histotype (Fig. 1b and c).

### Kaplan-Meier analysis reveals the prognostic value of COL3A1, GPR158 and PITHD1 protein expression

**Fig. 2:**
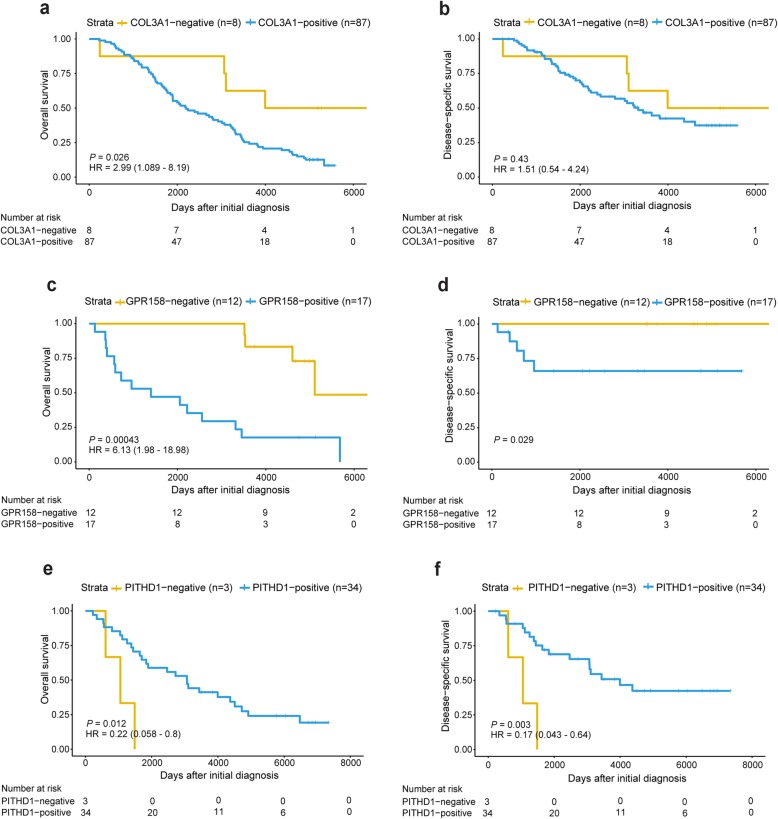
Kaplan-Meier survival analysis for COL3A1, GPR158 and PITHD1. Kaplan-Meier plots illustrating the probability of OS and DSS according to dichotomized protein expression of COL3A1 (a-b), GPR158 (c-d) and PITHD1 (e-f). Patients with COL3A1-positive protein expression revealed an association with shorter OS (*P* value = 0.026). GRP158-positive and PITHD1-negative protein expression showed both significantly shorter OS and DSS times (GPR158 OS *P* value = 0.00043, DSS *P* value 0.029; PITHD1 OS *P* value = 0.012, DSS *P* value = 0.003). The x-axes depict OS or DSS and the y-axes depict days after initial diagnosis. Hazard ratio (HR), 95% confidence interval, log rank *P* value were calculated using Cox proportional hazard model and log-rank tests, respectively

Kaplan-Meier curves and log-rank tests were used to estimate patient survival in relation to COL3A1, GPR158 and PITHD1 protein expression levels. The Kaplan-Meier curves were dichotomized according to the H-score cutoff for positive immunostaining. Kaplan-Meier analysis revealed an association between COL3A1, GPR158 and PITHD1 protein expression with OS and/or DSS (Fig. 2). More specifically, COL3A1 expression was associated with shorter OS rates (*P* value = 0.026, HR = 2.99 (95% CI 1.089–8.19)) in epithelial ovarian carcinoma patients. A significant association was not found for COL3A1 expression and DSS. Furthermore, GPR158 expression was associated with shorter OS and DSS (*P* value = 0.00043, HR = 6.13 (95% CI 1.98–18.98); *P* value = 0.029) in MC patients. Lastly, PITHD1 expression was associated with longer OS and DSS (*P* value = 0.012, HR = 0.22 (95% CI 0.058–0.80); *P* value = 0.003, HR = 0.17 (95% CI 0.043–0.64)) in CCC patients.

### Predictive performance is improved when combining COL3A1, GPR158 and PITHD1 expression with established clinicopathological parameters

Univariate and multivariable survival analyses for OS and DSS were performed to evaluate whether COL3A1, GPR158 and PITHD1 expression could improve outcome prediction. When adjusted for established clinical parameters (age, stage, CA125, ploidy and/or histotype), COL3A1 expression improved outcome prediction for OS (HR = 3.2, *P* value = 0.032), whereas the predictive performance of GPR158 and PITHD1 expression was improved for OS (HR = 23.83, *P* value = 0.00011), and OS and DSS (HR = 0.082, *P* value = 0.0013; HR = 0.047, *P* value = 0.00036) (Table 3). However, the improvement in outcome prediction was most prominent for GPR158 and PITHD1. The predictive model for GPR158 and OS showed the highest increase in C-index from 0.627 for established parameters to 0.795 in combination with GPR158 protein status (Additional file 3: Figure S3).

**Table 3:** Univariable and multivariable survival analysis for COL3A1, GPR158 and PITHD1 expression and overall and disease-specific survival. COL3A1 is adjusted for histotype, age, stage, CA125 and ploidy. GPR158 and PITHD1 are adjusted for age, stage, CA125 and ploidy. Significant values are marked in bold

	Overall survival			Disease-specific survival		
	HR (95% CI)	*P* value	C-index	HR (95% CI)	*P* value	C-index
Univariable analysis						
Histotype	–	0.55	0.541	**–**	**0.020**	**0.620**
Patient age	**1.018**	**0.038**	**0.568**	0.99	0.48	0.530
Stage	1.11	0.67	0.512	**2.0**	**0.016**	**0.583**
CA125	–	0.25	0.550	**–**	0.69	0.533
Ploidy	–	0.06	0.561	**–**	0.36	0.551
COL3A1	**2.99**	**0.026**	**0.537**	1.51	0.43	0.520
Multivariable analysis						
COL3A1	**3.20**	**0.032**	**0.654**	1.40	0.56	0.704
Univariable analysis						
Patient age	1.02	0.18	0.591	0.98	0.46	0.598
Stage	1.07	0.89	0.450	4.57	0.068	0.684
CA125	–	0.97	0.493	–	0.45	0.642
Ploidy	–	0.59	0.569	–	0.23	0.689
GPR158	**6.13**	**0.00043**	**0.690**	–	**0.029**	**0.748**
Multivariable analysis						
GPR158	**23.83**	**0.00011**	**0.795**	–	1.0	0.946
Univariable analysis						
Patient age	1.021	0.22	0.569	1.014	0.48	0.548
Stage	1.04	0.94	0.505	0.73	0.68	0.522
CA125	–	0.57	0.558	–	0.22	0.601
Ploidy	–	0.23	0.542	–	0.12	0.560
PITHD1	**0.22**	**0.012**	**0.558**	**0.17**	**0.003**	**0.574**
Multivariable analysis						
PITHD1	**0.082**	**0.0013**	**0.694**	**0.047**	**0.00036**	**0.733**

### KM plotter confirms the prognostic value of *COL3A1*, *GPR158* and *PITHD1* gene expression

**Fig. 3:**
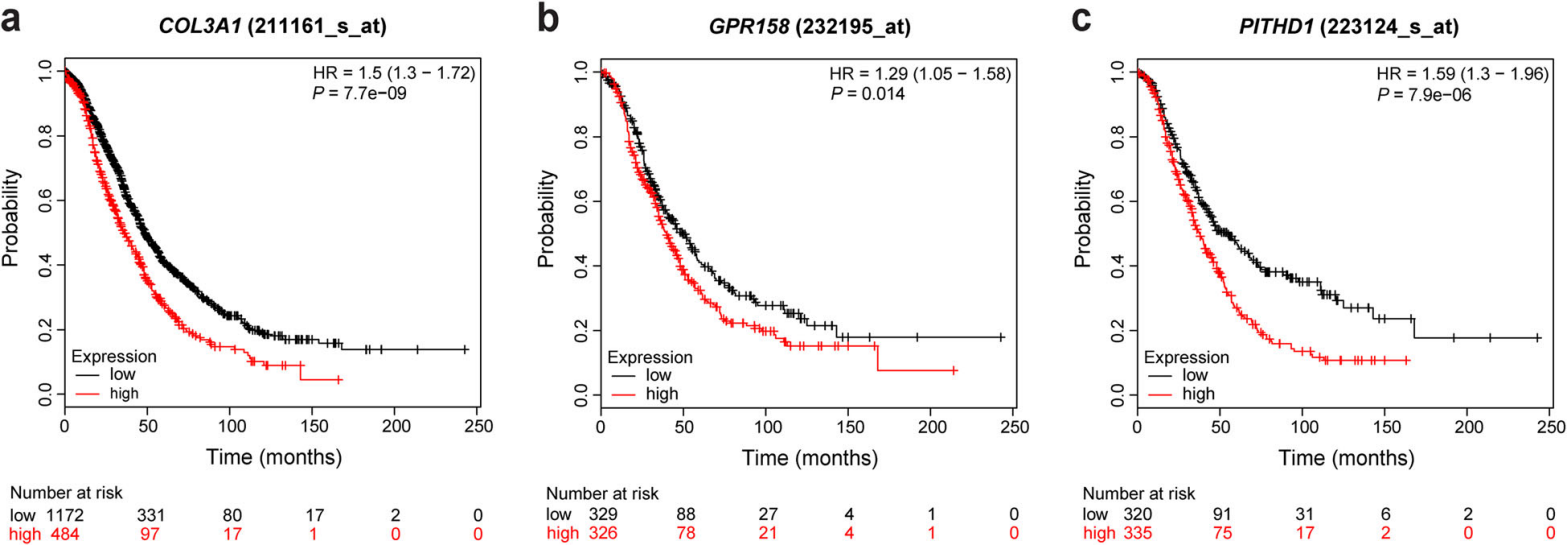
Validation of prognostic value of *COL3A1*, *GPR158* and *PITHD1* using KM plotter. Kaplan-Meier plots showing overall survival in HGSC and EC for a) *COL3A1* (*n* = 1656 patients), b) *GPR158* (*n* = 655 patients) and b) *PITHD1* (*n* = 655 patients). Red: patient samples with expression levels above the median, black: patient samples with expression levels below the median. *P* values less than 0.05 were considered significant. Number-at-risk is indicated below the main plot. Hazard ratio (HR), 95% confidence interval, log rank *P* value were calculated using Cox proportional hazard model and log-rank tests, respectively

KM plotter was used to validate the prognostic value of *COL3A1*, *GPR158* and *PITHD1* in an external ovarian cancer dataset. The Kaplan-Meier curves were dichotomized according to the median value of expression, wherein patient samples with expression levels above the median were placed in the high expression group and patient samples with expression levels below the median were classified in the low expression group. Fig. 3 shows significant Kaplan-Meier plots for *COL3A1* (Affymetrix ID: 211161_s_at, *n* = 1656 patients, *P* value = 7.7e-09), *GPR158* (Affymetrix ID: 232195_at, *n* = 655 patients, *P* value = 0.014) and *PITHD1* (Affymetrix ID: 223124_s_at, *n* = 655 patients, *P* value = 7.9e-06). All three genes showed significant differences in gene expression levels between the expression groups. High levels of *COL3A1*, *GPR158* and *PITHD1* expression correlated with lower patient survival rates. Furthermore, *COL3A1* (Affymetrix ID: 201852_x_at, *P* value = 2.1e-05; Affymetrix ID: 215076_s_at, *P* value = 3.8e-06) and *PITHD1* (Affymetrix ID: 223123_s_at, *P* value = 0.043) were also significantly associated with OS using the KM plotter (Additional file 4: Figure S4). A similar correlation between *GPR158* and *PITHD1* expression with lower OS were also obtained when tested in relation to OS for EC patients (GPR158: Affymetrix ID: 232195_at, *n* = 30 patients, *P* value = 0.063; PITHD1: Affymetrix ID: 223124, *n* = 30 patients, *P* value = 0.0004). Moreover, 21/26 study genes were significantly associated with patient survival for at least one Affymetrix ID probe (Additional file 7: Table S3). The FRMPD2, SLC9A4 and TRIM71 genes are not included on the GeneChip™ Human Genome U133A 2.0 Array and could therefore not be assessed with the KM plotter.

## Discussion

In the current study, the prognostic role of 29 genes was assessed in early-stage ovarian carcinomas using IHC. The patient samples used for biomarker validation represented a large cohort of early-stage ovarian carcinoma specimens(*n* = 206) distributed across four histotypes (HGSC (*n* = 94), EC (*n* = 46), MC (*n* = 29) and CCC (*n* = 37)). The *MTUS1* and *COL3A1* genes were chosen from our previous study [13] and the remaining 27 genes showed a promising correlation between RNA-seq expression and survival rates (OS and DSS) in different histotypes (HGSC, EC, MC and CCC). Unfortunately, antibodies for all of the 29 proteins could not be optimized, in part, due to potential discrepancies between transcript and protein expression levels, too low gene expression or unsuitable antibodies. RNA expression is a suitable indicator for protein level, but it does not always directly correlate with protein expression due to e.g. post transcriptional modification, translational and protein degradation regulation [29, 30]. It has been reported that intratumoral heterogeneity has limited impact on gene expression profiling and can be easily detected using IHC [31, 32].

Survival analysis revealed that the RNA expression levels for the *COL3A1*, *GPR158* and *PITHD1* genes were significantly correlated with that shown on the protein expression levels, proposing them as prognostic factors for ovarian carcinoma (COL3A1) and in MC (GPR158) and CCC histotypes (PITHD1). COL3A1 is part of the collagen family which may affect the tumor microenvironment to promote tumor progression by regulating the extracellular matrix via collagen degradation and re-deposition [33, 34]. GPR158 is an orphan class C member belonging to the G Protein coupled receptor (GPCR) superfamily of cell-surface signaling proteins which have been shown to exhibit aberrant expression in multiple cancers such as colon, breast, prostate and ovarian cancer, thereby contributing to e.g. tumor progression and metastasis [35, 36]. Furthermore, GPCRs are involved in many physiological and disease processes, making them important therapeutic targets [37]. This is reflected by GPCRs being the target of about 34% of all FDA-approved drugs [38]. Little is known about PITHD1. A recent study reported that PITHD1 is downregulated in leukemia and may regulate RUNX1 expression that promotes megakaryocyte differentiation, and activates the internal ribosomal entry site [39].

The IHC results showed that COL3A1 protein staining was mainly localized in the cytoplasm of tumor cells, but at varying intensity in the 95 RNA sequenced samples. Kruskal-Wallis analysis of variance also indicated that COL3A1 expression was dependent on histotype with lower expression in CCC patients. Previously, a variation in COL3A1 protein staining has been reported in e.g. epithelial tumor cells, in the cytoplasm but also in the nucleus of colorectal carcinoma, using 13,548–1-AP antibody from Proteintech [40]. As reported here, GPR158 was mainly localized in the cytoplasm of tumor cells, with occasional staining in the nuclei of tumor cells. The HPA database reports cytoplasmic, membranous and nuclear IHC staining for GPR158 in various tissues. Moreover, PITHD1 was herein primarily observed in tumor nuclei. Apart from a recent report where PITHD1 was detected in the cytoplasm of leukemic cells, little information is known about PITHD1 protein staining [39]. Similar expression levels for GPR158 and PITHD1 were observed on the RNA and protein levels. For COL3A1, higher RNA expression was demonstrated in comparison with protein expression in the whole cohort and in relation to each histotype. The lower COL3A1 protein expression may be explained by processes such as post transcriptional modification, translational and protein degradation regulation [29, 30].

The Kaplan-Meier analysis of dichotomized COL3A1 protein expression revealed an association with shorter OS, which is consistent with *COL3A1* RNA expression shown in our previous work [13]*.* A recent report identified *COL3A1* in a 7-gene signature related to stage in SC patients contributing to extracellular matrix interactions and mitosis [41], supporting our findings that COL3A1 may be a promising prognostic factor for ovarian tumor progression. Moreover, COL3A1, COL5A2 and COL1A2 expression are associated with drug-resistance in ovarian cancer [42]. However, the exact role of COL3A1 in ovarian tumorigenesis is yet to be revealed. COL3A1 has also been reported in other cancer types such as breast and colorectal cancers, with an association between *COL3A1* and *P4HA2* with poor prognosis in breast cancer, and correlation between elevated epithelial COL3A1 protein expression and unfavorable outcome in colorectal cancer [40, 43]. It has further been shown that Col3 suppresses metastatic processes of triple-negative breast cancer cells as well as tumor growth and metastasis in mice [44].

Survival analysis of GPR158-positive protein expression in MC patients showed an association with unfavorable OS which is consistent with that found on RNA expression level. Surprisingly, none of the MC ovarian carcinoma patients classified in the GPR158-negative expression group died of ovarian carcinoma. GPR158 has previously been shown to be involved in prostate cancer growth and progression, wherein up-regulation of GPR158 in Pten homozygous knock-out mice could contribute to tumor invasion [45]. Moreover, GPR158 has been shown to promote glioma stem cell differentiation and apoptosis [45, 46]. To our knowledge, no association has previously been shown for GPR158 expression and ovarian cancer. However, other genes of the GPCR family, such as GRP137 has been shown to be highly expressed in ovarian cancer tissue and gene knockdown resulted in a decrease in cell proliferation rates and inhibition of cell migration capabilities [47]. Furthermore, nuclear expression of GPR30 has been reported to be a negative prognostic factor for OS in epithelial ovarian cancer [48]. Expression of GPR56 in ovarian serous carcinoma was shown to be associated with advanced FIGO stage and to promote progression and invasion of epithelial ovarian cancer [49]. Therefore, GPR158 may present a novel prognostic factor for MC patients and may constitute a promising novel therapeutic target.

CCC patients with negative PITHD1 protein expression were associated with both significantly shorter OS and DSS. These results are not in line with that found on the RNA level, where elevated *PITHD1* expression was correlated with an unfavorable outcome. This contradiction may be due to the different techniques used, e.g. RNA-seq takes into account the transcriptomic expression of all cell types in the tumor specimen (e.g. tumor and stromal cells) whereas immunohistochemistry was scored solely using protein expression patterns in tumor cells. Hence, the discrepancy between PITHD1 RNA and protein levels may be due to the contribution of PITHD1 expression in stromal cells. To our knowledge, no association between PITHD1 expression and ovarian carcinoma has previously been shown and may therefore present a novel prognostic predictor for tumor progression in CCC patients.

Furthermore, combined predictive models containing protein expression status (COL3A1, GPR158 or PITHD1) together with established clinical parameters improved outcome prediction (increased C-index values) compared with models containing established clinical parameters alone, supporting the importance of these biomarkers. The results were further validated in an external cohort using the KM plotter database. RNA expression for all three genes were validated wherein an elevated RNA expression for *COL3A1*, *GPR158* and *PITHD1* correlated with unfavorable outcome. However, it should be noted that the majority of the samples in KM plotter datasets were advanced stage (III + IV) in the HGSC or EC histotypes. Unfortunately, there are currently no public databases comprising gene expression data for CCC or MC patients.

## Conclusions

In summary, we have validated three promising prognostic biomarkers on the protein level in ovarian carcinoma. COL3A1 may play an oncogenic role in epithelial ovarian carcinoma (HGSC, EC, MC, CCC), GPR158 in MC and PITHD1 in CCC, wherein COL3A1 and GPR158 protein expression act as predictors of unfavorable prognosis, whereas PITHD1 protein expression is associated with a favorable prognosis. Our results are interesting in terms of not only prognosis but also tumor progression. The knowledge from this study may in the future ideally be used to assist physicians in prognostication of ovarian carcinoma at the time of diagnosis. Further investigation using e.g. larger patient cohorts, and in vitro and in vivo models are needed to further validate the clinical and biological significance of these biomarkers in ovarian carcinoma histotypes.

## Additional files

Additional file 1: **Figure S1.** Variation of protein staining intensity in ovarian carcinoma. Pie charts representing the proportion of samples with weak, intermediate or strong staining intensities for each protein. Staining intensities of weak to strong are colored light blue, blue and dark blue. Eight of the nine tumor samples with strong COL3A1 intensity were of HGSC histotype. (TIF 848 kb)

Additional file 2: **Figure S2.** Variation in protein expression with regard to histotype and OS. COL3A1 protein expression differed depending on histotype (Additional file 2: Figure S2a) as well as histotype within the 5–10 year survival group (Additional file 2: **Figure S2b**. The x-axes depict COL3A1 H-score and the y-axes depict ovarian carcinoma histotype and survival time, wherein the patients have been stratified into four survival groups 0–2 years, 2–5 years, 5–10 years and > 10 years. (TIF 991 kb)

Additional file 3: **Figure S3.** Multivariable survival analysis for OS and DSS. The addition of the protein expression status resulted in improved outcome prediction for COL3A1 (a, b), GPR158 (c, d), PITHD1 (e, f). COL3A1 survival analysis was adjusted for histotype, age, stage, CA125, ploidy, and GPR158 and PITHD1 were adjusted for age, stage, CA125, ploidy. The x-axes depict C-index for OS or DSS and the y-axes depict survival time in days. C-index values for each outcome prediction curve are shown in parentheses. (TIF 2852 kb)

Additional file 4: **Figure S4.** Additional Affymetrix probes for validating *COL3A1* and *PITHD1* prognostic value using KM plotter. Kaplan-Meier plots showing overall survival in HGSC and EC for a-b) *COL3A1* (*n* = 1656 patients), and c) *PITHD1* (*n* = 655 patients). Red: patient samples with expression levels above the median, black: patient samples with expression levels below the median. *P* values less than 0.05 were considered significant. Number-at-risk is indicated below the main plot. Hazard ratio (HR), 95% confidence interval, log rank *P* were calculated using Cox proportional hazard model and log-rank tests. (TIF 1050 kb)

Additional file 5: **Table S1.** Reporting recommendations for tumor marker prognostic 642 studies (REMARK) guidelines. (DOCX 22 kb)

Additional file 6: **Table S2.** Distribution of clinicopathological characteristics 645 in relation to COL3A1, GPR158 and PITHD1 protein expression. (DOCX 22 kb)

Additional file 7: **Table S3.**. KM plotter probe ID and *P* values for the study 647 genes. Twenty-six of the 29 study genes could be evaluated using KM plotter. The *FRMPD2*, *SLC9A4* and *TRIM71* genes could not be assessed since they are not included on the GeneChip™ Human Genome U133A 2.0 Array. Twenty one genes showed significant Kaplan-Meier plots for at least one Affymetrix ID probe (marked in bold). (DOCX 26 kb)

## Data Availability

Raw RNA-seq read counts for 95 of the 206 ovarian tumors have been deposited in the NCBI Gene Expression Omnibus (http://www.ncbi.nlm.nih.gov/geo/) under accession number GSE101109.

## Abbreviations

95% CI: 95% Confidence Interval
AQP1: Aquaporin-1
AUC(t): Time-dependent Area under the receiver operating characteristics curve
CA 125: Cancer antigen 125
CCC: Clear-cell ovarian carcinoma
C-index: Concordance index
COL3A1: Collagen type III alpha 1 chain
CT 45: Cancer antigen 45
DSS: Disease-specific survival
EC: Endometrioid ovarian carcinoma
FDR: False discovery rate
FFPE: Full-face formalin-fixed paraffin-embedded
FIGO: International Federation of Gynecology and Obstetrics
GEO: Gene Expression Omnibus
GPR158: G protein-coupled receptor 158
HE4: Human epididymis protein 4
HGSC: High-grade serous ovarian carcinoma
HPA: Human Protein Atlas
HR: Hazard ratio
H-score: Immunoreactive score
IHC: Immunohistochemistry
MC: Mucinous ovarian carcinoma
MIA: Multivariate index assay
OS: Overall survival
PITHD1: PITH domain containing 1
RNA-seq: RNA sequencing
SC: Serous ovarian carcinoma
TCGA: The Cancer Genome Atlas
