# Supplementary figures and images for: Immunohistochemical validation of COL3A1, GPR158 and PITHD1 as prognostic biomarkers in early-stage ovarian carcinomas
Source: BMC Cancer. 2019 Sep 18;19:928. doi: 10.1186/s12885-019-6084-4 (PMC6751742; doi:10.1186/s12885-019-6084-4)

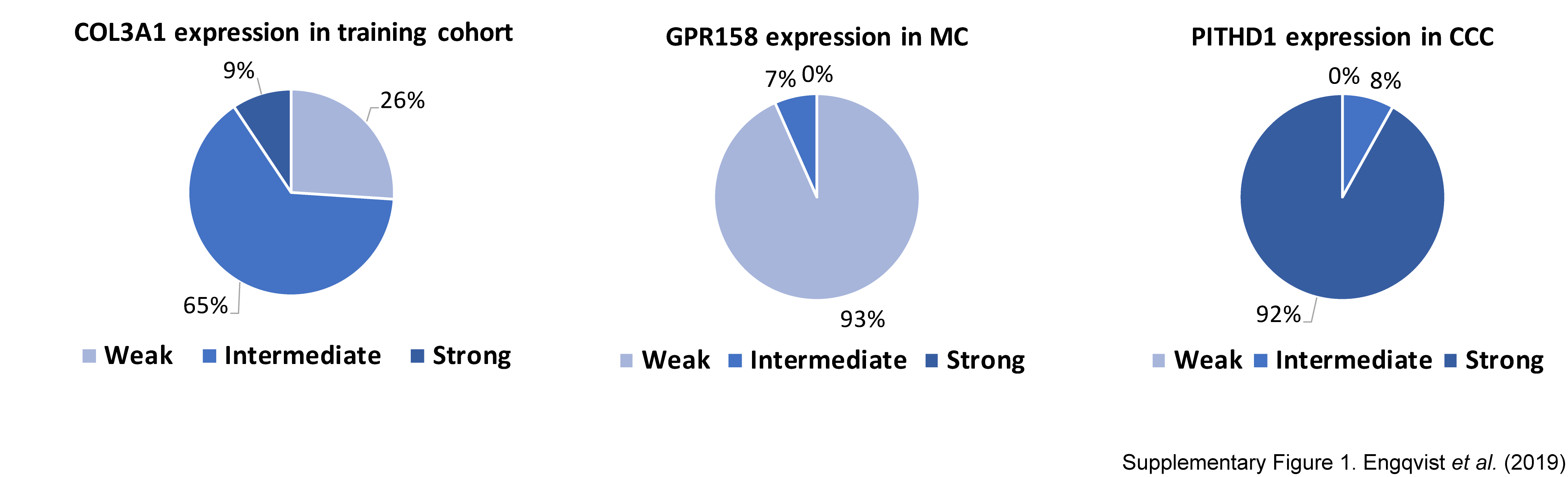

Supplement: Supplementary file 1 — Figure S1. Variation of protein staining intensity in ovarian carcinoma. Pie charts representing the proportion of samples with weak, intermediate or strong staining intensities for each protein. Staining intensities of weak to strong are colored light blue, blue and dark blue. Eight of the nine tumor samples with strong COL3A1 intensity were of HGSC histotype. (TIF 848 kb) [file 12885_2019_6084_MOESM1_ESM.tif]

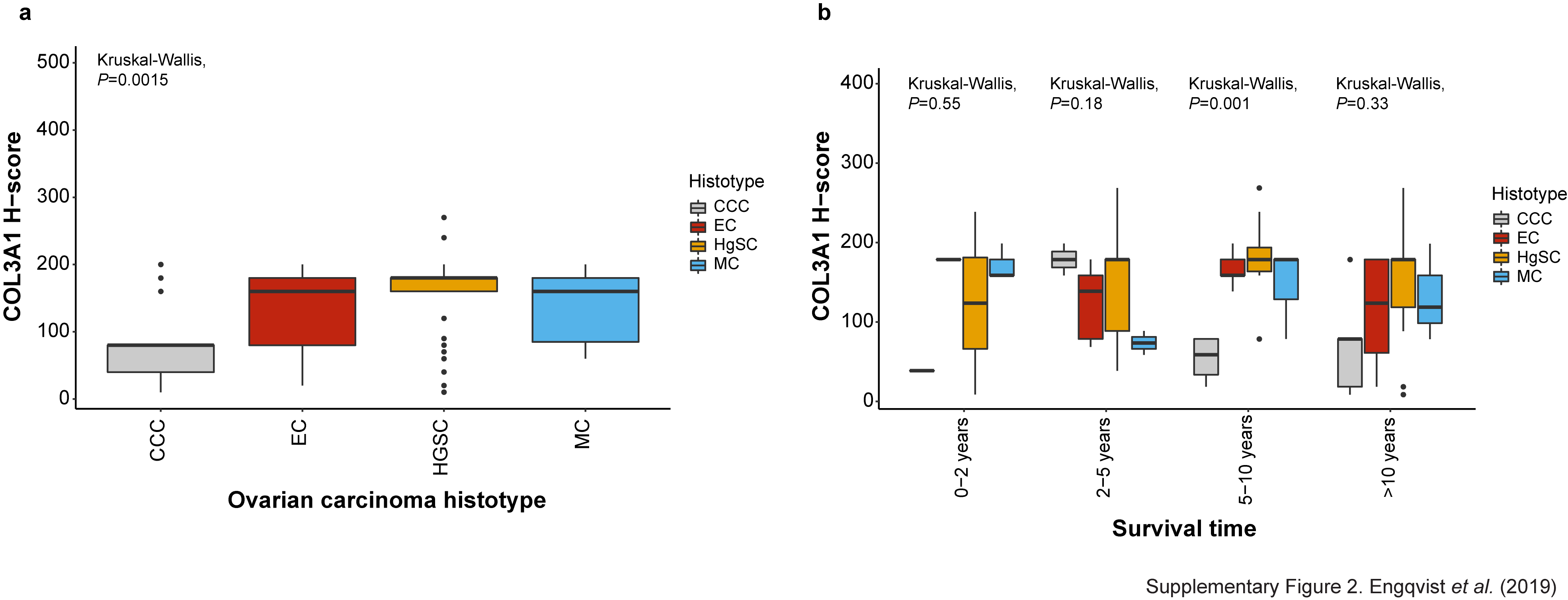

Supplement: Supplementary file 2 — Figure S2. Variation in protein expression with regard to histotype and OS. COL3A1 protein expression differed depending on histotype (Additional file 2: Figure S2a) as well as histotype within the 5–10 year survival group (Additional file 2: Figure S2b. The x-axes depict COL3A1 H-score and the y-axes depict ovarian carcinoma histotype and survival time, wherein the patients have been stratified into four survival groups 0–2 years, 2–5 years, 5–10 years and > 10 years. (TIF 991 kb) [file 12885_2019_6084_MOESM2_ESM.tif]

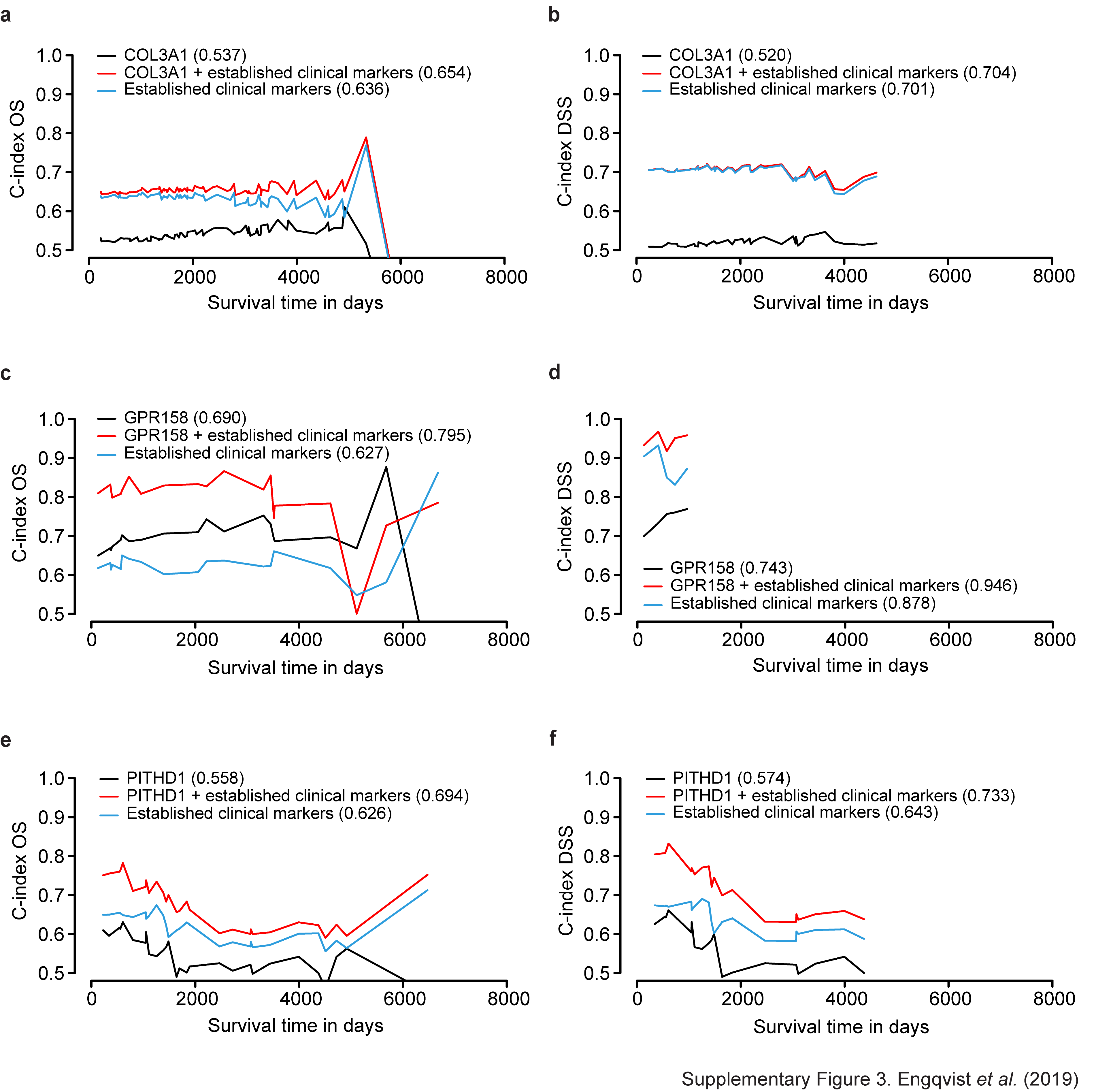

Supplement: Supplementary file 3 — Figure S3. Multivariable survival analysis for OS and DSS. The addition of the protein expression status resulted in improved outcome prediction for COL3A1 (a, b), GPR158 (c, d), PITHD1 (e, f). COL3A1 survival analysis was adjusted for histotype, age, stage, CA125, ploidy, and GPR158 and PITHD1 were adjusted for age, stage, CA125, ploidy. The x-axes depict C-index for OS or DSS and the y-axes depict survival time in days. C-index values for each outcome prediction curve are shown in parentheses. (TIF 2852 kb) [file 12885_2019_6084_MOESM3_ESM.tif]

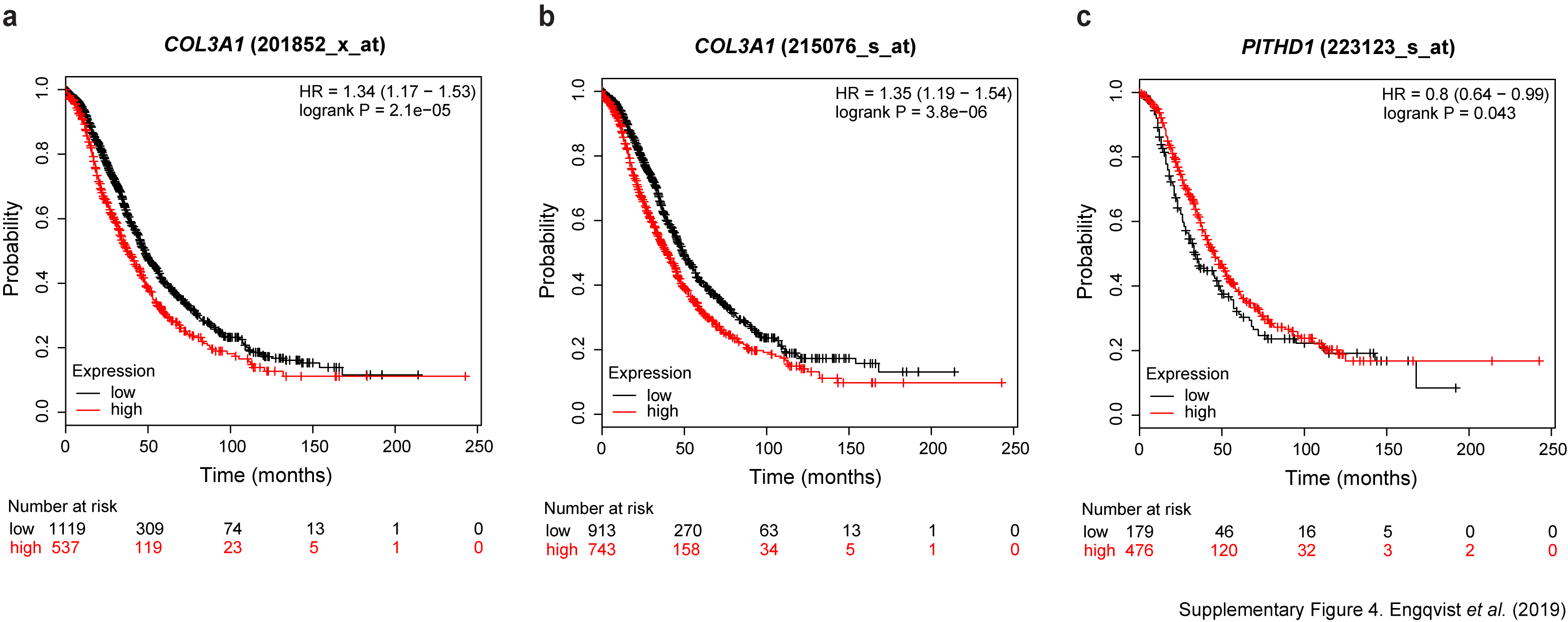

Supplement: Supplementary file 4 — Figure S4. Additional Affymetrix probes for validating COL3A1 and PITHD1 prognostic value using KM plotter. Kaplan-Meier plots showing overall survival in HGSC and EC for a-b) COL3A1 (n = 1656 patients), and c) PITHD1 (n = 655 patients). Red: patient samples with expression levels above the median, black: patient samples with expression levels below the median. P values less than 0.05 were considered significant. Number-at-risk is indicated below the main plot. Hazard ratio (HR), 95% confidence interval, log rank P were calculated using Cox proportional hazard model and log-rank tests. (TIF 1050 kb) [file 12885_2019_6084_MOESM4_ESM.tif]
