# Supplementary material for: Immunohistochemical validation of COL3A1, GPR158 and PITHD1 as prognostic biomarkers in early-stage ovarian carcinomas
Source: BMC Cancer. 2019 Sep 18;19:928. doi: 10.1186/s12885-019-6084-4 (PMC6751742; doi:10.1186/s12885-019-6084-4)
Supplement: Supplementary file 5 — Table S1. Reporting recommendations for tumor marker prognostic 642 studies (REMARK) guidelines. (DOCX 22 kb) [file 12885_2019_6084_MOESM5_ESM.docx]

**Additional file Table S1. Reporting recommendations for tumor marker prognostic studies (REMARK) guidelines.**

| **Item to be reported** | |
| --- | --- |
| **INTRODUCTION** | |
| 1 | *State the marker examined, the study objectives, and any pre-specified hypotheses.*  Immunohistochemistry (IHC) was used to validate the clinical significance of 29 prognostic biomarkers identified using RNA sequencing (RNA-seq) for early-stage ovarian carcinoma.  Examined markers:  Study cohort: ARHGAP21, ARMC3, C7, CDH18, CES3, COL3A1, COL11A1, EHD3, FRMPD2, GABRP, GID4, GPR158, GRM5, IGHG1, JCHAIN, KIF26B, MAP7D2, MTRNR2L1, MTUS1, MUC15, PITHD1, PTEN, RTKN2, SLC9A4, SMYD2, TRIM71, TRIO, TTK, and VNN1 (Table 2).  Established markers: patient age, stage, CA125, ploidy and/or histotype. |
| **MATERIALS AND METHODS** | |
| *Patients* | |
| 2 | *Describe the characteristics (e.g., disease stage or co-morbidities) of the study patients, including their source and inclusion and exclusion criteria.*  The clinicopathological characteristics of the ovarian carcinoma patients included in the cohort are described in “Patients and tumor samples” in the Methods section and Table 1. |
| 3 | *Describe treatments received and how chosen (e.g., randomized or rule-based).*  Treatment protocols were based on national treatment guidelines. All patients underwent laparotomy and debulking cytoreductive surgery. The majority of patients (197/206) received adjuvant therapy with chemotherapy (Table 1). |
| *Specimen characteristics* | |
| 4 | *Describe type of biological material used (including control samples) and methods of preservation and storage.*  Full-face formalin-fixed paraffin-embedded (FFPE) specimens obtained from the Department of Clinical Pathology at Sahlgrenska University Hospital (Gothenburg, Sweden) for 206 early-stage (stage I and II) primary invasive ovarian carcinoma patients, diagnosed between 1994 and 2006, were used in the study. One sample in the optimization panel, consisting of 15 full-face FFPE ovarian carcinoma sections representing varying histotypes (HGSC, EC, MC, CCC) and FIGO stages, was chosen as positive control for each immunohistochemical experiment. |
| *Assay methods* | |
| 5 | *Specify the assay method used and provide (or reference) a detailed protocol, including specific reagents or kits used, quality control procedures, reproducibility assessments, quantitation methods, and scoring and reporting protocols. Specify whether and how assays were performed blinded to the study endpoint.*  Two pathologists, who were blinded to the survival data, performed the microscopic analysis of immunostained tissue sections. An immunoreactive score (H-score) was calculated for each tumor specimen based on the percentage and intensity of positively stained tumor cells, where 0 = negative, 1 = weak positive, 2 = moderate positive and 3 = strong positive staining. The H-score values ranged between 0 and 300, where H-score equaled (1 x %1) + (2 x %2) + (3 x %3). |
| *Study design* | |
| 6 | *State the method of case selection, including whether prospective or retrospective and whether stratification or matching (e.g., by stage of disease or age) was used. Specify the time period from which cases were taken, the end of the follow-up period, and the median follow-up time.*  The ovarian carcinoma patients (n=206) were retrospectively selected from biobanks at the Departments of Clinical Pathology at hospitals in Western Sweden. The patients were diagnosed between 1994 and 2006. Clinicopathological characteristics and overall survival data were obtained from the National Quality Registry at the Regional Cancer Center West (Gothenburg, Sweden) and the Cancer Registry at the National Board of Health and Welfare. Patients were chosen for inclusion in the study cohort according to early-stage ovarian carcinomas (FIGO stage I and II), overall survival calculated from the date of initial diagnosis to the date of death of any cause and stratified into four survival groups, i.e. 0-2 years, 2-5 years, 5-10 years and >10 years, as well as histotype (HGSC, EC, CCC, MC). Furthermore, the patient was only included if the stored FFPE sample were a primary invasive ovarian carcinoma.  The end of follow-up-period was October 2015 and the median follow-up time was 3089 days. |
| 7 | *Precisely define all clinical endpoints examined.*  Overall survival (OS) was defined as the time from initial diagnosis to death from any cause, and disease-specific survival (DSS) was defined as the time from initial diagnosis to ovarian cancer-related death. |
| 8 | *List all candidate variables initially examined or considered for inclusion in models.*  Multivariable Cox proportional hazard models were used to assess the predictive strength (C-index) of three of the study cohort proteins (COL3A1, GPR158 and PITHD1) when adjusted by established clinical parameters (age, stage, CA125, ploidy and/or histotype). |
| 9 | *Give rationale for sample size; if the study was designed to detect a specified effect size, give the target power and effect size.*  A total of 206 samples were included in the patient cohort of which 94 high-grade serous ovarian carcinomas (HGSC), 46 endometrioid (EC), 29 mucinous (MC), and 37 clear-cell ovarian carcinomas (CCC) samples. The sample size provided sufficient statistical power. |
| *Statistical analysis methods* | |
| 10 | *Specify all statistical methods, including details of any variable selection procedures and other model-building issues, how model assumptions were verified, and how missing data were handled.*   1. *Study cohort selection*   “Selection of study genes” in the Methods section describes the selection of the 29 promising prognostic biomarkers.   1. *Association of marker values/histotypes with clinicopathological characteristics*   The R-package tableone (v. 0.9.3) was used to calculate possible confounding factors (Table 1, Supplementary Table 1).   1. *Patient survival estimation in relation to protein expression levels*   Kaplan-Meier curves were generated and tested with log rank tests using survival time (OS/DSS) and dichotomized H-score for positive immunostaining (survival v. 2.40-1 and survminer v. 0.4.3) (Figure 2).   1. *Methods to evaluate the biomarker’s univariable and multivariable association with clinical outcome*   Univariable and multivariable Cox proportional hazard models were used to assess the predictive strength (C-index) of COL3A1, GPR158 and PITHD1 expression in relation to OS and DSS when adjusted by established clinical parameters (age, stage, CA125, ploidy and/or histotype) (Table 3).   1. *Comparison between RNA and protein expression*   The R package ggplot2 (v. 3.1.0) and Kruskal-Wallis test were used to generate box plots to compare RNA-protein expression and differences in H-score between the histotype and survival groups.   1. *External validation*   The Kaplan-Meier plotter online tool (KM plotter, http://kmplot.com/analysis/) for ovarian cancer was used to determine the clinical relevance of gene expression for the study genes in relation to overall survival   1. *Missing data*   In the survival analysis, patients with missing values (Table 1, “Not available”) were excluded. |
| 11 | *Clarify how marker values were handled in the analyses; if relevant, describe methods used for cutpoint determination.*  The H-score was used to correlate the protein expression levels to overall survival and disease-specific survival. An H-score cutoff stratifying the tumor specimens in positive and negative protein expression was determined for each study gene using Kaplan-Meier plots in X-tile Software (v. 3.6.1) (23). |
| **RESULTS** | |
| *Data* | |
| 12 | *Describe the flow of patients through the study, including the number of patients included in each stage of the analysis (a diagram may be helpful) and reasons for dropout. Specifically, both overall and for each subgroup extensively examined report the numbers of patients and the number of events.*  The number of patients used in the different analyses is detailed in Table 1 and 2. |
| 13 | *Report distributions of basic demographic characteristics (at least age and sex), standard (disease-specific) prognostic variables, and tumour marker, including numbers of missing values.*  The clinicopathological characteristics including numbers of missing values for the 206 patients are listed in Table 1. |
| *Analysis and presentation* | |
| 14 | *Show the relation of the marker to standard prognostic variables.*  The relationship between prognostic markers and established clinicopathological data is shown in Supplementary Figure 3 and Table 3. |
| 15 | *Present univariable analyses showing the relation between the marker and outcome, with the estimated effect (e.g., hazard ratio and survival probability). Preferably provide similar analyses for all other variables being analysed. For the effect of a tumour marker on a time-to-event outcome, a Kaplan-Meier plot is recommended.*  Kaplan-Meier plots and univariable analyses including hazard ratio, *P* value and C-index for COL3A1, GPR158 and PITHD1, are presented in Figure 2 and Table 3. |
| 16 | *For key multivariable analyses, report estimated effects (e.g., hazard ratio) with confidence intervals for the marker and, at least for the final model, all other variables in the model.*  Multivariable analyses including hazard ratio, *P* value and C-index for COL3A1, GPR158 and PITHD1 are stated in Table 3. |
| 17 | *Among reported results, provide estimated effects with confidence intervals from an analysis in which the marker and standard prognostic variables are included, regardless of their statistical significance.*  Table 3 describes univariable and multivariable analyses including established clinical parameters (age, stage, CA125, ploidy and/or histotype). |
| 18 | *If done, report results of further investigations, such as checking assumptions, sensitivity analyses, and internal validation.*  The results of the external validation are detailed in the Results section. |
| **DISCUSSION** | |
| 19 | *Interpret the results in the context of the pre-specified hypotheses and other relevant studies; include a discussion of limitations of the study.*  The study results were discussed in the context of pre-specified hypotheses and other relevant studies in the Discussion section. |
| 20 | *Discuss implications for future research and clinical value.*  Further investigation using *e.g.* larger patient cohorts, and *in vitro* and *in vivo* models could further validate the clinical and biological significance of the examined biomarkers in ovarian carcinoma histotypes. COL3A1 may play an oncogenic role in epithelial ovarian carcinoma (HGSC, EC, MC, CCC), GPR158 in MC patients and PITHD1 in CCC patients, wherein COL3A1 and GPR158 protein expression act as predictors of unfavorable prognosis, whereas PITHD1 protein expression is associated with a favorable prognosis. |
