# Supplementary material for: Immunohistochemical validation of COL3A1, GPR158 and PITHD1 as prognostic biomarkers in early-stage ovarian carcinomas
Source: BMC Cancer. 2019 Sep 18;19:928. doi: 10.1186/s12885-019-6084-4 (PMC6751742; doi:10.1186/s12885-019-6084-4)
Supplement: Supplementary file 6 — Table S2. Distribution of clinicopathological characteristics 645 in relation to COL3A1, GPR158 and PITHD1 protein expression. (DOCX 22 kb) [file 12885_2019_6084_MOESM6_ESM.docx]

**Additional file Table S2. Distribution of clinicopathological characteristics in relation to COL3A1, GPR158 and PITHD1 protein expression.**
