# Supplementary material for: Immunohistochemical validation of COL3A1, GPR158 and PITHD1 as prognostic biomarkers in early-stage ovarian carcinomas
Source: BMC Cancer. 2019 Sep 18;19:928. doi: 10.1186/s12885-019-6084-4 (PMC6751742; doi:10.1186/s12885-019-6084-4)
Supplement: Supplementary file 7 — Table S3.. KM plotter probe ID and P values for the study 647 genes. Twenty-six of the 29 study genes could be evaluated using KM plotter. The FRMPD2, SLC9A4 and TRIM71 genes could not be assessed since they are not included on the GeneChip™ Human Genome U133A 2.0 Array. Twenty one genes showed significant Kaplan-Meier plots for at least one Affymetrix ID probe (marked in bold). (DOCX 26 kb) [file 12885_2019_6084_MOESM7_ESM.docx]

**Additional file Table S3. KM plotter probe ID and *P* values for the study genes.** Twenty-six of the 29 study genes could be evaluated using KM plotter. The *FRMPD2*, *SLC9A4* and *TRIM71* genes could not be assessed since they are not included on the GeneChip™ Human Genome U133A 2.0 Array. Twenty-one genes showed significant Kaplan-Meier plots for at least one Affymetrix ID probe (marked in bold).
